# Prevalence of use and real‐world effectiveness of smoking cessation aids during the COVID‐19 pandemic: a representative study of smokers in England

**DOI:** 10.1111/add.15903

**Published:** 2022-05-13

**Authors:** Sarah E. Jackson, Sharon Cox, Lion Shahab, Jamie Brown

**Affiliations:** ^1^ Department of Behavioural Science and Health University College London London UK; ^2^ SPECTRUM Consortium UK

**Keywords:** behavioural support, COVID‐19, e‐cigarettes, effectiveness, nicotine replacement therapy, quit attempts, smoking cessation, varenicline

## Abstract

**Aim:**

To measure whether the prevalence of use and real‐world effectiveness of different smoking cessation aids has changed in England since the coronavirus disease 2019 (COVID‐19) pandemic.

**Design:**

Representative monthly cross‐sectional surveys, January 2015–June 2021.

**Setting:**

England.

**Participants:**

A total of 7300 adults (≥18 y) who had smoked within the previous 12 months and had made ≥1 quit attempt during that period.

**Measurements:**

The independent variable was the timing of the COVID‐19 pandemic (pre‐pandemic [January 2015–February 2020] vs pandemic [April 2020–June 2021]). We analysed (i) the association between the pandemic period and self‐reported use (vs non‐use) during the most recent quit attempt of: prescription medication (nicotine replacement therapy [NRT]/varenicline/bupropion), NRT bought over‐the‐counter, e‐cigarettes, traditional behavioural support and traditional remote support (telephone support/written self‐help materials/websites) and (ii) the interaction between the pandemic period and use of these cessation aids on self‐reported abstinence from quit date to survey. Covariates included age, sex, social grade, level of cigarette addiction and characteristics related to the quit attempt.

**Findings:**

After adjustment for secular trends, there was a significant increase from the pre‐pandemic to pandemic period in the prevalence of use of traditional remote support by past‐year smokers in a quit attempt (OR = 2.18; 95% CI, 1.42–3.33); specifically telephone support (OR = 7.16; 95% CI, 2.19–23.45) and websites (OR = 2.39; 95% CI, 1.41–4.08). There was also an increase in the prevalence of use of prescription medication (OR = 1.47; 95% CI, 1.08–2.00); specifically varenicline (OR = 1.66; 95% CI, 1.09–2.52). There were no significant changes in prevalence of use of other cessation aids after adjustment for secular trends. People who reported using prescription medication (OR = 1.41; 95% CI, 1.09–1.84) and e‐cigarettes (OR = 1.87; 95% CI, 1.62–2.16) had greater odds of reporting abstinence than people who did not. There were no significant interactions between the pandemic period and use of any cessation aid on abstinence, after adjustment for covariates and use of the other aids, although data were insensitive to distinguish no change from meaningful modest (OR = 1.34) effects (Bayes factors 0.72–1.98).

**Conclusions:**

In England, the COVID‐19 pandemic was associated with an increase in use of remote support for smoking cessation and varenicline by smokers in a quit attempt up to June 2021. The data were inconclusive regarding an association between the pandemic and changes in the real‐world effectiveness of popular smoking cessation aids.

## INTRODUCTION

Smoking prevalence in England has declined steadily over the past decade [1], and the government has set an ambitious goal to be smoke free by 2030 [2], but this will not be achieved without increasing the quit rate [3]. Approximately 30% of smokers typically report making an attempt to quit smoking permanently each year and 5% (~20% of those who try) successfully stop smoking in the short‐to‐medium term (up to a year after the quit attempt) [4]. A range of cessation aids are effective in increasing the odds that a given quit attempt is successful [5, 6, 7, 8, 9, 10, 11, 12, 13, 14]. Under usual circumstances, these are widely available in England, at little or no cost, to smokers who want to quit. However, restrictions introduced to control the coronavirus disease 2019 (COVID‐19) pandemic altered the way in which many of these cessation aids could be accessed, which may have affected their uptake and/or effectiveness for cessation. Increases in quitting activity have been documented among smokers in England during the first COVID‐19 lockdown, with the rate of past‐year quit attempts rising to around 40% and the overall quit rate to 9% (from quit date to time of survey) [15]. To understand changes in quitting activity during the pandemic, it is important to evaluate the extent to which smokers' use of different cessation aids—and the effectiveness of these aids in supporting abstinence—has changed during the COVID‐19 pandemic. These data will provide useful insight into how progress toward achieving the government's smoke free target may be affected by the pandemic.

England has among the most extensive and comprehensive coverage, as well as highest rates of use, of medications and behavioural support for smoking cessation in the world [16]. Pharmacological options include nicotine replacement therapy (NRT), which can be bought over‐the‐counter (OTC) or obtained free of charge or cheaply on prescription and prescription‐only medications, varenicline (Champix) and bupropion (Zyban). E‐cigarettes are most commonly purchased from specialist ‘vape shops’ [17] and are also available in supermarkets, smaller convenience stores and online. Dedicated stop smoking services commissioned by local authorities offer a combination of free behavioural support and pharmacotherapy. Some also offer e‐cigarette starter kits and many welcome people who want to receive support while using an e‐cigarette [18]. A free Smokefree National Helpline is available for telephone support, and national and regional websites provide information on cessation and how to access other forms of support. Around half of quit attempts by smokers in England are supported by the use of one or more of these cessation aids [19]. The most popular aid is e‐cigarettes (used in ~30% of quit attempts), followed by NRT OTC (~10%) and prescription medication (~6%) [19]. Uptake of remote cessation aids (e.g. websites) that can be accessed from home is low (~3%) [20].

The United Kingdom (UK) government responded to the COVID‐19 pandemic by implementing a national lockdown with work, travel and social contact restrictions enforceable by law on 23 March 2020. This had an immediate impact on the ways in which smokers were able to access support for quitting. Vape shops were classed as ‘non‐essential retail’ and were forced to close; online shops remained open [21]. This saw a shift in source of purchase for vaping devices and e‐liquids, with sales growth in supermarkets and convenience stores (which lack the specialist knowledge offered by vape shops) rising more than 20% [22]. Stop smoking services, which traditionally operate a face‐to‐face delivery model, had to rapidly adapt to provide remote support. A survey of stop smoking service providers found the most widely used method of providing advice to smokers during lockdown was telephone advice (98% of services surveyed); 58% used video conferencing [23]. There are some advantages to remote service delivery, including flexibility of scheduling appointments, users not needing to travel and users preferring the more informal/less clinical approach [23]. However, concerns were also raised that a remote delivery model might exclude smokers who lack access to information technology and make it more difficult to engage certain groups (e.g. smokers from deprived areas or ethnic minorities) [23, 24]. Pharmacies remained open throughout lockdown, but it is possible that some smokers did not consider NRT an essential purchase that warranted leaving home. General practitioners (GPs) remained open, but switched to remote appointments. Many people found it difficult to book a GP appointment at times during the pandemic and struggled to get medication they required [25], meaning smokers may have been less able to access prescription medications to support cessation. Non‐essential shops reopened on 15 June 2020 and restrictions were gradually eased during July and August, but varied regionally according to levels of transmission. During this time, support provision varied: although vape shops were open as usual, only 18% of stop smoking services were offering face‐to‐face advice in August and September 2020 [23]. Two further lockdowns were implemented in November 2020 and between January and April 2021 with similar restrictions on non‐essential shops and public services.

It is possible that the changes in smokers' access to popular forms of cessation support brought about by the COVID‐19 pandemic may have affected the prevalence of their use in a quit attempt. We previously analysed changes in smoking and quitting outcomes during the first month of lockdown in England [15]. Results indicated that there had been no significant change in use of evidence‐based support (defined as NRT OTC, prescription medication, face‐to‐face behavioural support or e‐cigarettes) compared with before the pandemic, but were suggestive of a potential increase in use of remote support (defined as telephone support, websites, or apps). However, the sample size recruited during lockdown was small (*n* < 200), limiting statistical power to explore changes in use of specific aids separately. With more data now available, it is possible to undertake a more detailed assessment of changes in use of cessation aids during the pandemic.

The pandemic may also have affected the real‐world effectiveness of popular cessation aids in helping smokers to quit successfully. For example, as the closure of vape shops drove consumers to purchase their supplies elsewhere, with less specialised support and a more limited range of products, there was also a shift in the types of devices being purchased away from open‐system devices to closed‐system pod devices [22]. Studies suggest closed‐system devices may be less effective for cessation [26, 27, 28], meaning this shift in device type purchases could have affected the real‐world effectiveness of e‐cigarettes during the pandemic. Alternatively, pharmacological support can be particularly effective at preventing lapses, and the greater exposure to stress during the pandemic [29] may have accentuated this advantage. Remote stop smoking services may have been more successful in recruiting and/or retaining smokers who would otherwise struggle to attend in‐person appointments, which could increase, reduce or have no effect on the real‐world effectiveness of such support depending on the treatment response of this group of smokers. In addition, if support is harder to access, self‐selection may increase estimates of treatment effectiveness, if those who are more motivated to quit are more likely to seek out support.

This study aimed to examine whether the prevalence of use and real‐world effectiveness of different smoking cessation aids has changed in England since the COVID‐19 pandemic. This information will provide important context around previous findings documenting a substantial increase in the success rate of quit attempts by smokers in England during the early stages of the pandemic [15, 30]. Specifically, we aimed to address the following research questions:

1. Among smokers making a quit attempt in England, to what extent has the prevalence of use of prescription medication (NRT, varenicline and bupropion), NRT OTC, e‐cigarettes, traditional behavioural support and traditional remote support (telephone support, written self‐help materials and websites) changed since the COVID‐19 pandemic, relative to the 5 years before the pandemic?
2. Among smokers making a quit attempt in England, has the association between use of the above cessation aids and success in the quit attempt been moderated by the COVID‐19 pandemic?

## METHOD

### Design and population

Data were drawn from the ongoing Smoking Toolkit Study, a monthly cross‐sectional survey of a representative sample of adults in England designed to provide insights into population‐wide influences on smoking and cessation by monitoring trends on a range of variables relating to smoking [31]. The study uses a form of random location sampling to select a new sample of ~1700 adults ages ≥16 years each month. Comparisons with sales data and other national surveys show that the Smoking Toolkit Study recruits a representative sample of the population in England with regard to key demographic variables, smoking prevalence and cigarette consumption [31, 32].

Data are usually collected monthly through face‐to‐face computer assisted interviews. However, social distancing restrictions under the COVID‐19 pandemic meant that no data were collected in March 2020 and data from April 2020 onward were collected via telephone, and the lower age bound for participation was increased from 16 to 18 years because of changes in consenting procedures. The telephone‐based data collection relied on the same combination of random location and quota sampling, and weighting approach as the face‐to‐face interviews and previous diagnostic analyses conducted on the first month of telephone data indicate good comparability between the two data collection modalities [15, 30].

For the present study, we used aggregated individual‐level data from respondents age ≥18 years (to match the age range of participants surveyed since the pandemic) to the survey in the period from January 2015 (during this time use of different cessation aids has been relatively stable following a rapid increase in use of e‐cigarettes) to June 2021 (the latest wave of the survey for which data were available at the time of analysis). Our sample comprised respondents who reported: (i) smoking cigarettes (including hand‐rolled) or any other tobacco product (e.g. pipe or cigar) daily or occasionally at the time of the survey or during the preceding 12 months (‘past‐year smokers’), and (ii) having made at least one quit attempt in the preceding 12 months, assessed with the question ‘How many serious attempts to stop smoking have you made in the past 12 months? By serious, I mean you decided that you would try to make sure you never smoked again.’

### Measures

#### Measurement of outcomes

Use of smoking cessation aids was assessed with the question: ‘Which, if any, of the following did you try to help you stop smoking during the most recent serious quit attempt?’ Respondents were asked to indicate all that apply, and data for each of the following were coded 1 if chosen and 0 if not: (i) prescription NRT (available in England from prescribing health professionals, including advisors at specialist stop smoking services); (ii) NRT OTC (NRT bought over the counter without a prescription); (iii) varenicline; (iv) bupropion; (v) e‐cigarettes; (vi) traditional behavioural support (attended a stop smoking group or attended one or more stop smoking one‐to‐one counselling/advice/support session[s], either face‐to‐face or remotely via video conferencing); (vii) telephone support (phoned a smoking helpline); (viii) written self‐help materials (a book or booklet); and (ix) websites. For analysis, we combined variables relating to prescription medication (prescription NRT, varenicline and bupropion) and traditional remote support (telephone support, written self‐help materials and websites) because we anticipated the numbers of participants reporting using these aids to be small [33]. Where sample sizes permitted, use of these aids were also analysed separately.

Successful smoking cessation was defined as self‐reported continuous abstinence from the start of the most recent quit attempt up to the time of survey. Respondents were asked ‘How long did your most recent quit attempt last before you went back to smoking?’ Responses were coded 1 for those who responded that they were still not smoking and 0 otherwise.

#### Measurement of independent variables

Timing of the COVID‐19 pandemic was coded 1 for April 2020 through June 2021 (survey waves 162–176; pandemic period) and 0 for January 2015 through February 2020 (waves 100–161; pre‐pandemic period).

#### Measurement of covariates

Sociodemographic covariates were age, sex and occupational social grade (ABC1, which includes managerial, professional and intermediate occupations, vs C2DE, which includes small employers and own‐account workers, lower supervisory and technical occupations and semi‐routine and routine occupations, state pensioners, never worked and long‐term unemployed). This occupational measure of social grade is a valid classification that is widely used in research in UK populations [34].

Level of cigarette addiction was assessed by self‐reported ratings of the strength of urges to smoke over the last 24 hours (not at all [coded 0], slight [1], moderate [2], strong [3], very strong [4], extremely strong [5]). This question was also coded ‘0’ for smokers who responded ‘not at all’ to the (separate) question ‘How much of the time have you spent with the urge to smoke?’ [35]. This measure has been validated and performs at least as well as the Fagerström Test of Cigarette Dependence and the Heaviness of Smoking Index in predicting the outcome of cessation while not being subject to bias because of population‐level changes in cigarette consumption over the time period of the study [36].

Variables relating to the most recent quit attempt were also included, including time since the quit attempt started, the number of prior quit attempts in the past year (categorised as 1, 2, 3 or ≥4), whether the quit attempt was planned or occurred immediately when the decision to quit was made and whether the respondent cut down first or stopped abruptly.

The month of survey was also included to take account of seasonal variation in quit attempts (e.g. in January or ‘Stoptober’).

### Statistical analysis

The analysis plan was pre‐registered on Open Science Framework (https://osf.io/y5c8w/). We made two amendments to the analyses following peer review: (i) we reran our analysis of changes in the prevalence of use of cessation aids during the pandemic period relative to the pre‐pandemic period adjusting for survey wave to take account of secular trends; and (ii) we added a sensitivity analysis in which we restricted the sample to those whose quit attempt began >6 months ago.

Data were analysed on complete cases using SPSS v.25. Characteristics of the samples recruited during the pre‐pandemic and pandemic periods were compared using Pearson's χ^2^ for categorical variables and one‐way analysis of variance for continuous variables.

The following analyses were conducted for the following categories of smoking cessation aids: prescription medication, NRT OTC, e‐cigarettes, traditional behavioural support and traditional remote support. Where it was feasible for a given sample size, we repeated these analyses separately for individual types of prescription medication (NRT, varenicline and bupropion) and traditional remote support (telephone support, written self‐help materials and websites).

We used logistic regression to compare the mean prevalence of use of each aid during the pandemic period relative to the pre‐pandemic period, with and without adjustment for survey wave (coded January 2015 = 1 through June 2021 = 78) to take account of underlying linear trends. We plotted the quarterly prevalence of use of each smoking cessation aid to show trends over the study period.

We then constructed a series of logistic regression models in which we tested the two‐way interaction between each cessation aid (use of a specific aid vs no use of that specific aid) and the timing of the pandemic (pre‐pandemic vs pandemic period) on abstinence (abstinent yes vs no). Each model was fully adjusted for all other cessation aids (to estimate the unique association between each cessation aid and abstinence) and the covariates outlined above. In an unplanned sensitivity analysis added following peer review, we repeated these analyses restricting the sample to smokers whose most recent quit attempt began more than 6 months ago to assess whether the pattern of results differed when we focused on longer‐term cessation.

Bayes factors (BF) were calculated to determine whether interaction results were supportive of the alternative hypothesis (i.e. the pandemic has moderated the real‐world effectiveness of popular cessation aids), the null hypothesis or were insensitive. We used a half‐normal distribution, the mode at 0 (no effect), and the SD equal to the expected effect size, which we set at OR = 1.34 (or equivalent in the observed direction, i.e. 0.75 for observed ORs <1) on the basis that this is the documented difference between using no cessation support and the lowest intensity effective cessation support [11] and would, therefore, constitute a meaningful change in effectiveness. As an unplanned sensitivity analysis, we also calculated BFs with the expected effect size set at OR = 3 to assess whether large changes in effectiveness could be ruled out. BFs ≥3 can be interpreted as evidence for the alternative hypothesis (and against the null), BFs ≤1/3 as evidence for the null hypothesis, and BFs between 1/3 and 3 suggest the data are insensitive to distinguish the alternative hypothesis from the null [37, 38].

## RESULTS

A total of 133 536 participants aged ≥18 years took part in the Smoking Toolkit Study between January 2015 and June 2021. Of these, 24 454 (18.3%) were past‐year smokers, of whom 7574 (32.0%) reported having made at least one serious attempt to quit in the past 12 months. We excluded 274 participants (3.6%) with missing data on one or more variables of interest. The analysed sample, therefore, comprised 7300 participants, 5750 (78.8%) of whom completed the survey before the pandemic (January 2015–February 2020) and 1550 (21.2%) during the pandemic (April 2020–June 2021).

Table 1 summarises sample characteristics in relation to the timing of the pandemic. Relative to those recruited in the pre‐pandemic period, past‐year smokers who attempted to quit in the last 12 months recruited during the pandemic were significantly more likely to be younger, from social grades ABC1, to report weaker urges to smoke (i.e. lower nicotine dependence), to have started their quit attempt more than 6 months earlier, not to have planned their quit attempt and to report still being abstinent at the time of the survey.

**TABLE 1:** Characteristics of the analysed samples of past‐year smokers attempting to quit smoking in the past 12 months who were recruited before and during the COVID‐19 pandemic

	*Pre‐pandemic (Jan 2015–Feb 2020)*	*Pandemic (Apr 2020–Jun 2021)*	*P*
*N*	5750	1550	–
Age, y			
18–24	18.4 (1056)	21.2 (328)	<0.001
25–34	23.8 (1371)	28.0 (434)	–
35–44	17.8 (1024)	17.0 (263)	–
45–54	16.7 (958)	14.0 (217)	–
55–64	12.9 (739)	10.5 (163)	–
≥ 65	10.5 (602)	9.4 (145)	–
Female	48.7 (2802)	50.0 (775)	0.375
Social grade C2DE	54.5 (3132)	49.3 (764)	<0.001
Strength of urges to smoke^a^, mean (SD)	1.80 (1.21)	1.55 (1.30)	<0.001
Time since quit attempt started			
<1 mo	17.3 (997)	12.1 (188)	<0.001
1–3 mo	24.5 (1409)	19.2 (297)	–
3–6 mo	21.3 (1226)	25.7 (399)	–
>6 mo	36.8 (2118)	43.0 (666)	–
No. of quit attempts in the past year			
1	66.3 (3811)	64.3 (997)	0.508
2	20.1 (1154)	21.2 (329)	–
3	7.1 (406)	7.7 (120)	–
4 or more	6.6 (379)	6.7 (104)	–
Planned attempt	47.9 (2754)	42.2 (654)	<0.001
Abrupt attempt (no cutting down first)	51.9 (2982)	53.2 (824)	0.363
Still abstinent at the time of the survey (overall quit rate)	16.7 (958)	26.2 (406)	<0.001

*Note*: Figures are presented as percentage (*n*), unless stated otherwise.

^a^ Strength of urges to smoke: 0 (no urges) to 5 (extremely strong urges).

### Research question 1: has the prevalence of use of cessation aids changed since the pandemic?

**FIGURE 1:**
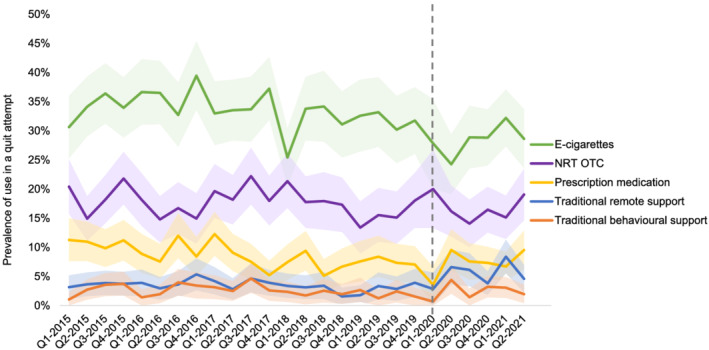
Quarterly prevalence of use of cessation aids by past‐year smokers in a quit attempt: January 2015–June 2021. The vertical grey line at Q1–2020 indicates the start of the first COVID‐19 lockdown in England. Shaded bands indicate the 95% confidence interval. NRT OTC, nicotine replacement therapy over‐the‐counter

**FIGURE 2:**
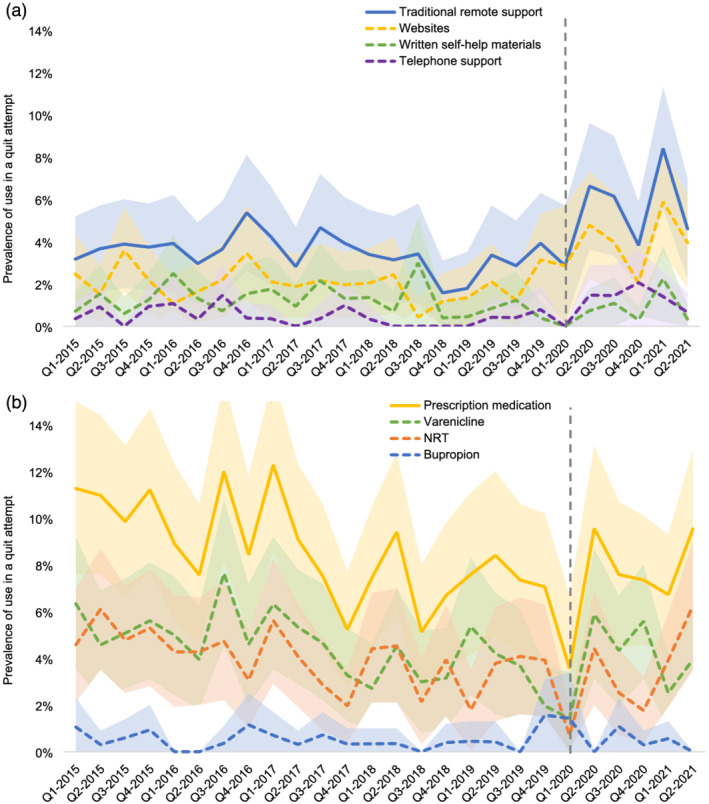
Quarterly prevalence of use of (a) remote cessation support options and (b) prescription medications by past‐year smokers in a quit attempt: January 2015–June 2021. The vertical grey line at Q1–2020 indicates the start of the first COVID‐19 lockdown in England. Shaded bands indicate the 95% confidence interval. NRT, nicotine replacement therapy

Table 2 shows the mean prevalence of use of each cessation aid in the pre‐pandemic and pandemic periods. Figures 1 and 2 show quarterly prevalence data across the study period.

**TABLE 2:** Prevalence of use of cessation aids before and during the COVID‐19 pandemic

	*Pre‐pandemic (Jan 2015–Feb 2020)*	*Pandemic (Apr 2020–Jun 2021)*	*OR [95% CI]*	*P*	*OR*_*adj*_ ^a^ *[95% CI*]	*P*
Prescription medication	8.6 (497)	8.1 (125)	0.93 [0.76–1.14]	0.469	1.47 [1.08–2.00]	0.015
NRT	4.0 (230)	3.7 (58)	0.93 [0.70–1.25]	0.643	1.45 [0.93–2.26]	0.102
Varenicline	4.5 (259)	4.4 (68)	0.97 [0.74–1.28]	0.843	1.66 [1.09–2.52]	0.019
Bupropion	0.5 (30)	0.4 (6)	0.74 [0.31–1.78]	0.503	0.69 [0.21–2.31]	0.547
NRT over‐the‐counter	17.9 (1028)	16.2 (251)	0.89 [0.76–1.03]	0.122	0.93 [0.75–1.16]	0.509
E‐cigarettes	33.4 (1923)	28.8 (446)	0.80 [0.71–0.91]	0.001	0.91 [0.46–1.08]	0.281
Traditional behavioural support	2.6 (147)	2.8 (44)	1.11 [0.79–1.57]	0.537	1.55 [0.91–2.63]	0.107
Traditional remote support	3.5 (200)	5.9 (92)	1.75 [1.36–2.26]	<0.001	2.18 [1.42–3.33]	<0.001
Telephone support	0.5 (26)	1.4 (22)	3.17 [1.79–5.61]	<0.001	7.16 [2.19–23.45]	0.001
Written self‐help materials	1.2 (69)	1.0 (15)	0.81 [0.46–1.41]	0.448	1.10 [0.49–2.47]	0.824
Websites	2.1 (118)	4.1 (64)	2.06 [1.51–2.80]	<0.001	2.39 [1.41–4.08]	0.001
None of the above (unaided quitting)	43.0 (2471)	51.0 (790)	1.38 [1.23–1.54]	<0.001	1.14 [0.97–1.35]	0.113

*Note*: Figures are presented as percentage (*n*), unless stated otherwise.

NRT, nicotine replacement therapy.

^a^ Adjusted for secular trend (survey wave: January 2015 = 1 through June 2021 = 78). A significant OR indicates a significant change in use, taking into account the underlying linear trend.

Relative to the pre‐pandemic period, there was an increase in the prevalence of use of traditional remote support (+68.6%) (Figure 1)—specifically telephone support and websites (+180.0% and +95.2%, respectively) (Figure 2a)—by past‐year smokers in a quit attempt. These changes were statistically significant before and after adjustment for the underlying trends in prevalence of use of these aids (Table 2).

Although simple pre‐post comparisons of the prevalence of use of prescription medications indicated little change during the pandemic, a significant increase in the prevalence of use of prescription medication (Figure 1)—specifically varenicline (Figure 2b)—was detected when we took into account underlying trends in these variables over the study period (Table 2).

Prescription NRT followed a similar pattern, but the increase did not reach statistical significance (Figure 2b, Table 2).

There was a reduction in the prevalence of use of e‐cigarettes (−13.8%) and an increase in unaided quitting (+18.6%), but these changes were largely attributable to a continuation of the underlying trends rather than step‐level changes in prevalence when the pandemic started (Figure 1). The prevalence of use of other cessation aids did not differ significantly in comparisons of the pre‐pandemic to pandemic periods.

### Research question 2: has the real‐world effectiveness of cessation aids changed since the pandemic?

There was no significant interaction between the timing of the pandemic and use of any cessation aid on abstinence, after adjustment for use of the other aids, sociodemographic characteristics, strength of urges to smoke and characteristics of the quit attempt (Table 3). BF indicated that the data were insensitive, meaning we could not distinguish modest changes (OR = 1.34) from no changes in the real‐world effectiveness of aids. Results were unchanged when positing a larger possible effect (OR = 3), except for e‐cigarettes and NRT OTC where a change in effectiveness of this size could be ruled out. We note that overall quit rates were significantly higher during the pandemic period relative to the pre‐pandemic period (Table 1), which may explain the appearance of higher effectiveness of all the cessation aids (with the exception of bupropion) in the unadjusted quit rates shown in Table 3.

**TABLE 3:** Interactions between timing of the COVID‐19 pandemic (pre‐pandemic vs pandemic) and use of cessation aids on abstinence

	*Quit rate (n/N)*		*OR*_*adj*_ ^a^ *[95% CI]*	*P*	*BF (OR = 1.34)*	*Interpretation of BF*	*BF (OR = 3)*	*Interpretation of BF*
	*Pre‐pandemic*	*Pandemic*						
Prescription medication	14.9% (74/497)	27.2% (34/125)	1.20 [0.67–2.13]	0.544	1.04	Data are insensitive	0.45	Data are insensitive
NRT	12.6% (29/230)	25.9% (15/58)	1.40 [0.60–3.26]	0.438	1.22	Data are insensitive	0.73	Data are insensitive
Varenicline	17.4% (45/259)	32.4% (22/68)	1.21 [0.57–2.56]	0.617	1.03	Data are insensitive	0.50	Data are insensitive
Bupropion^b^	13.3% (4/30)	0.0% (0/6)	–	–	–	–	–	–
NRT over‐the‐counter	13.2% (136/1028)	19.9% (50/251)	0.95 [0.61–1.48]	0.824	0.72	Data are insensitive	0.24	Moderate evidence for H0
E‐cigarettes	20.0% (384/1923)	28.3% (126/446)	0.91 [0.65–1.26]	0.563	0.78	Data are insensitive	0.25	Moderate evidence for H0
Traditional behavioural support	11.6% (17/147)	29.5% (13/44)	2.32 [0.85–6.32]	0.101	1.93	Data are insensitive	2.39	Data are insensitive
Traditional remote support	12.0% (24/200)	26.1% (24/92)	1.78 [0.85–3.77]	0.129	1.98	Data are insensitive	1.67	Data are insensitive
Telephone support	11.5% (3/26)	36.4% (8/22)	4.50 [0.80–25.14]	0.087	1.55	Data are insensitive	2.78	Data are insensitive
Written self‐help materials	13.0% (9/69)	20.0% (3/15)	1.10 [0.21–5.80]	0.908	0.97	Data are insensitive	0.66	Data are insensitive
Websites	13.6% (16/118)	23.4% (15/64)	1.07 [0.43–2.66]	0.890	0.90	Data are insensitive	0.44	Data are insensitive

BF, Bayes factor; OR_adj_, adjusted odds ratio; NRT, nicotine replacement therapy; H0, null hypothesis.

Note: results reflect the interaction between use (vs non‐use) of each cessation aid and the timing of the pandemic on odds of abstinence.

^a^ Adjusted for use of all other aids, age, sex, social grade, strength of urges to smoke, time since the quit attempt started (≤6 months vs >6 months), number of past‐year quit attempts, whether the quit attempt was planned, and whether the quit attempt was abrupt or gradual. ORs >1 indicate increased effectiveness of the aid during the pandemic vs pre‐pandemic, and ORs <1 indicate reduced effectiveness.

^b^ An interaction could not be computed for bupropion because of inadequate sample size.

Table 4 summarises associations between use of each cessation aid and abstinence. Over the entire study period, use of e‐cigarettes and prescription medication—specifically varenicline—in a quit attempt was significantly associated with increased odds of still being abstinent at the time of the survey. Other aids were not significantly associated with increased odds of successful cessation.

**TABLE 4:** Association between use of cessation aids and abstinence, January 2015–June 2021

	*Quit rate (n/N)*	*OR*_*adj*_ *[95% CI]*	*P*
Prescription medication	17.4% (108/622)	1.41 [1.09–1.84]	0.010
NRT	15.3% (44/288)	1.34 [0.91–1.96]	0.140
Varenicline	20.5% (67/327)	1.59 [1.14–2.21]	0.006
Bupropion	11.1% (4/36)	0.94 [0.31–2.82]	0.904
NRT over‐the‐counter	14.5% (186/1279)	1.10 [0.90–1.33]	0.347
E‐cigarettes	21.5% (510/2369)	1.87 [1.62–2.16]	<0.001
Traditional behavioural support	15.7% (30/191)	1.12 [0.69–1.80]	0.654
Traditional remote support	16.4% (48/292)	0.89 [0.62–1.28]	0.528
Telephone support	22.9% (11/48)	1.10 [0.48–2.50]	0.828
Written self‐help materials	14.3% (12/84)	0.68 [0.34–1.36]	0.274
Websites	17.0% (31/182)	0.99 [0.63–1.57]	0.976

OR_adj_, adjusted odds ratio; NRT, nicotine replacement therapy.

Note: results reflect the odds of abstinence associated with use (vs non‐use) of each cessation aid, adjusted for use of all other aids, age, sex, social grade, strength of urges to smoke, time since the quit attempt started (≤6 months vs >6 months), number of past‐year quit attempts, whether the quit attempt was planned and whether the quit attempt was abrupt or gradual.

Results were not substantially altered when we restricted the sample to smokers whose quit attempts began more than 6 months before the survey (Supplementary Tables 1 and 2).

## DISCUSSION

Respondents to the survey during the COVID‐19 pandemic (April 2020–June 2021) were significantly more likely than those surveyed before the pandemic (January 2015–February 2020) to report using traditional remote support for smoking cessation, in particular telephone support and websites. They were also significantly more likely to report using prescription medication, particularly varenicline. Across the entire study period, use of e‐cigarettes and varenicline were associated with the highest odds of successful cessation. No significant difference in real‐world effectiveness during the pandemic relative to before the pandemic was observed for any cessation aid, although the data were insensitive.

During the early stages of the pandemic in England, there were marked increases both in the proportion of smokers who made a quit attempt and the proportion that were successful in achieving abstinence [15]. Given lockdown restrictions required changes to the delivery and accessibility of certain types of cessation support, it was plausible that the increased rate of quit success could be attributed to a shift in the types of cessation aids being used by smokers during the pandemic (with increased uptake of those known to be more effective) or a change in the real‐world effectiveness of these aids. We explored these potential explanations in the present analyses.

In terms of changes in prevalence of use, we found that despite potential barriers to accessing many of the commonly used aids (e.g. difficulty booking a GP appointment [25] or stop smoking services stopping in‐person support) [23], use of prescription medication—specifically varenicline—increased, and there was no significant change in the use of NRT OTC or traditional behavioural support. Use of traditional remote support increased—in particular, telephone support and websites. However, although the prevalence of use of these remote aids roughly doubled, absolute prevalence remained low (telephone: 1.4%; websites: 4.1%). E‐cigarettes remained the most commonly used cessation aid despite vape shops closing during periods of lockdown. Although there was a reduction in the proportion of smokers using e‐cigarettes in a quit attempt during the pandemic, this change was not statistically significant when we adjusted for the secular trend and likely reflects a continued decline in use that pre‐dated the pandemic.

In terms of changes in the real‐world effectiveness of cessation aids, the data were inconclusive. Although we observed no significant interaction between the pandemic and use of cessation aids on abstinence at the time of the survey, sample sizes for many of the aids were small and BF indicated the data were insensitive. What was clear, however, was that the rate of quit success increased regardless of which cessation aid was used, suggesting the increased rate of quitting observed in previous studies [15] may be related to the pandemic per se (e.g. changes in the profile of those who tried to quit—people appeared to be less dependent, younger and more likely to be from a higher social grade; increased motives to remain abstinent in the context of a respiratory virus; fewer social and environmental cues that trigger relapse; the financial impact of furlough/job loss necessitating reduced spending on cigarettes), rather than any effects of the pandemic on cessation support.

Further data on the real‐world effectiveness of popular cessation aids during the pandemic would be useful for informing decisions on future provision of services. For example, if a switch to offering remote services did increase the effectiveness of traditional behavioural support offered by stop smoking services—or even if was equally effective—a hybrid service could provide the best of both worlds in being able to support smokers unwilling or unable to access a remote service while allowing those who find it difficult to meet in person to use the remote offering. With both the government and public health experts emphasising a desire to ‘build back better’ following the pandemic [39, 40], including a focus on digital support [41, 42], now is an opportune moment to consider the optimal delivery of support to smokers who want to quit.

This study had several limitations. First, variability in restrictions during the pandemic period might mean short‐term changes during the periods of tightest restriction were obscured. We present quarterly data on prevalence of the use of the various cessation aids to offer some insight into shorter‐term changes, but small samples precluded more granular estimates of the real‐world effectiveness of the aids. Second, measurement of quit attempts referred to the most recent quit attempt that occurred during the past year. Therefore, for some of the participants surveyed during the pandemic period, the quit attempt could have taken place before the start of the pandemic. This could have led us to underestimate associations between the pandemic and our outcomes of interest. Third, adherence to participants' chosen cessation aid(s) was not assessed, so we cannot comment on whether this changed during the pandemic. Finally, sample sizes were relatively small for certain cessation aids, reducing statistical power. BFs indicated the data were insensitive to detect anything other than large significant associations between the pandemic and real‐world effectiveness of cessation aids, meaning we were unable to draw firm conclusions on this research question.

In conclusion, in England, the COVID‐19 pandemic was associated with an increase in use of remote support for smoking cessation and varenicline by smokers in a quit attempt up to June 2021. The data were inconclusive regarding an association between the pandemic and changes in the real‐world effectiveness of popular smoking cessation aids.

## ETHICS APPROVAL AND CONSENT TO PARTICIPATE

Ethical approval for the STS was granted originally by the UCL Ethics Committee (ID 0498/001). The data are not collected by UCL and are anonymized when received by UCL.

## DECLARATION OF INTERESTS

J.B. has received unrestricted research funding from Pfizer, who manufacture smoking cessation medications. L.S. has received honoraria for talks, an unrestricted research grant and travel expenses to attend meetings and workshops from Pfizer and has acted as paid reviewer for grant awarding bodies and as a paid consultant for health care companies. All authors declare no financial links with tobacco companies or e‐cigarette manufacturers or their representatives.

## AUTHOR CONTRIBUTIONS

**Sarah Jackson:** Conceptualization; formal analysis; investigation; methodology; visualization. **Sharon Cox:** Conceptualization; investigation; methodology. **Lion Shahab:** Conceptualization; funding acquisition; investigation; methodology. **Jamie Brown:** Conceptualization; data curation; funding acquisition; investigation; methodology; resources; supervision.

## Supporting information


**Table S1** Interactions between timing of the COVID‐19 pandemic (pre‐pandemic vs pandemic) and use of cessation aids on abstinence: sensitivity analysis restricting the sample to smokers whose quit attempt began >6 months ago
**Table S2** Association between use of cessation aids and abstinence, January 2015–June 2021: sensitivity analysis restricting the sample to smokers whose quit attempt began >6 months ago
