# Supplementary material for: Prevalence of use and real‐world effectiveness of smoking cessation aids during the COVID‐19 pandemic: a representative study of smokers in England
Source: Addiction. 2022 May 13;117(9):2504–14. doi: 10.1111/add.15903 (PMC9347508; doi:10.1111/add.15903)
Supplement: Supplementary file 1 — Table S1 Interactions between timing of the COVID‐19 pandemic (pre‐pandemic vs pandemic) and use of cessation aids on abstinence: sensitivity analysis restricting the sample to smokers whose quit attempt began >6 months ago Table S2 Association between use of cessation aids and abstinence, January 2015–June 2021: sensitivity analysis restricting the sample to smokers whose quit attempt began >6 months ago [file ADD-117-2504-s001.docx]

# Prevalence of use and real-world effectiveness of smoking cessation aids during the Covid-19 pandemic: a representative study of smokers in England

Supplementary material

| **Supplementary Table 1.** Interactions between timing of the Covid-19 pandemic (pre-pandemic vs. pandemic) and use of cessation aids on abstinence: sensitivity analysis restricting the sample to smokers whose quit attempt began >6 months ago | | | | | | | | | |
| --- | --- | --- | --- | --- | --- | --- | --- | --- | --- |
|  | | **Quit rate (n/N)** | | **OR_adj_ [95% CI]** | ***p*** | **BF (OR=1.34)** | **Interpretation of BF** | **BF**  **(OR=3)** | **Interpretation of BF** |
|  |  | **Pre-pandemic** | **Pandemic** |  |  |  |  |  |  |
| Prescription medication | | 14.3% (30/210) | 17.6% (9/51) | 0.63 [0.22-1.78] | 0.381 | 1.27 | Data are insensitive | 0.92 | Data are insensitive |
|  | NRT | 10.6% (10/94) | 25.0% (6/24) | 1.42 [0.33-6.18] | 0.641 | 1.07 | Data are insensitive | 0.79 | Data are insensitive |
|  | Varenicline | 16.8% (18/107) | 15.4% (4/26) | 0.46 [0.11-1.99] | 0.301 | 1.29 | Data are insensitive | 1.30 | Data are insensitive |
|  | Bupropion* | 13.3% (2/15) | 0%  (0/4) | - | - | - | - | - | - |
| NRT over-the-counter | | 12.0% (44/366) | 19.8% (19/96) | 0.89 [0.41-1.92] | 0.762 | 0.93 | Data are insensitive | 0.42 | Data are insensitive |
| E-cigarettes | | 21.6% (148/686) | 30.8% (62/201) | 0.96 [0.56-1.63] | 0.872 | 0.75 | Data are insensitive | 0.27 | Moderate evidence for H0 |
| Traditional behavioural support | | 12.3% (7/57) | 20.0% (4/20) | 2.02 [0.36-11.19] | 0.422 | 1.18 | Data are insensitive | 1.12 | Data are insensitive |
| Traditional remote support | | 14.1% (9/64) | 31.7% (13/41) | 2.57 [0.77-8.57] | 0.126 | 1.68 | Data are insensitive | 2.18 | Data are insensitive |
|  | Telephone support* | 0%  (0/5) | 45.5% (5/11) | - | - | - | - | - | - |
|  | Written self-help materials | 18.2% (4/22) | 22.2% (2/9) | 1.82 [0.18-18.56] | 0.615 | 1.07 | Data are insensitive | 0.99 | Data are insensitive |
|  | Websites | 12.5% (5/40) | 25.9% (7/27) | 0.97 [0.21-4.58] | 0.971 | 0.95 | Data are insensitive | 0.60 | Data are insensitive |
| BF, Bayes factor; CI, confidence interval; OR_adj_, adjusted odds ratio; NRT, nicotine replacement therapy.  *An interaction could not be computed for bupropion or telephone support due to inadequate sample size.  Note: results reflect the interaction between use (vs. non-use) of each cessation aid and the timing of the pandemic on odds of abstinence, adjusted for use of all other aids, age, sex, social grade, strength of urges to smoke, number of past-year quit attempts, whether the quit attempt was planned, and whether the quit attempt was abrupt or gradual. ORs >1 indicate increased effectiveness of the aid during the pandemic vs. pre-pandemic, and ORs <1 indicate reduced effectiveness. | | | | | | | | | |

| **Supplementary Table 2.** Association between use of cessation aids and abstinence, January 2015-June 2021: sensitivity analysis restricting the sample to smokers whose quit attempt began >6 months ago | | | | |
| --- | --- | --- | --- | --- |
|  | | **Quit rate (n/N)** | **OR_adj_ [95% CI]** | ***p*** |
| Prescription medication | | 14.9% (39/261) | 1.22 [0.78-1.91] | 0.393 |
|  | NRT | 13.6% (16/118) | 1.54 [0.80-2.97] | 0.197 |
|  | Varenicline | 16.5% (22/133) | 1.07 [0.60-1.93] | 0.816 |
|  | Bupropion | 10.5% (2/19) | 0.78 [0.16-3.89] | 0.764 |
| NRT over-the-counter | | 13.6% (63/462) | 0.91 [0.64-1.27] | 0.569 |
| E-cigarettes | | 23.7% (210/887) | 1.99 [1.56-2.54] | <0.001 |
| Traditional behavioural support | | 14.3% (11/77) | 1.11 [0.49-2.52] | 0.796 |
| Traditional remote support | | 21.0% (22/105) | 1.02 [0.56-1.86] | 0.945 |
|  | Telephone support | 31.3% (5/16) | 1.39 [0.37-5.21] | 0.627 |
|  | Written self-help materials | 19.4% (6/31) | 0.71 [0.24-2.07] | 0.526 |
|  | Websites | 17.9% (12/67) | 0.96 [0.44-2.10] | 0.912 |
| CI, confidence interval; OR_adj_, adjusted odds ratio; NRT, nicotine replacement therapy.  Note: results reflect the odds of abstinence associated with use (vs. non-use) of each cessation aid, adjusted for use of all other aids, age, sex, social grade, strength of urges to smoke, number of past-year quit attempts, whether the quit attempt was planned, and whether the quit attempt was abrupt or gradual. | | | | |
